# Bypassing use-dependent plasticity in the primary motor cortex to preserve adaptive behavior

**DOI:** 10.1038/s41598-021-91663-9

**Published:** 2021-06-08

**Authors:** M. Bosc, G. Bucchioni, B. Ribot, T. Michelet

**Affiliations:** 1 Univ. Bordeaux, CNRS, IMN, UMR 5293, 33000 Bordeaux, France; 2 Cold Spring Harbor Laboratory, Cold Spring Harbor, NY USA; 3 Univ. Bordeaux, CNRS, EPHE, INCIA, UMR 5287, 33000 Bordeaux, France; 4 iBrain, UMR 1253 Inserm, Université de Tours, 2 Boulevard Tonnellé, 37044 Tours Cedex, France

**Keywords:** Cognitive control, Motor cortex

## Abstract

Behavioral adaptation, a central feature of voluntary movement, is known to rely on top-down cognitive control. For example, the conflict-adaptation effect on tasks such as the Stroop task leads to better performance (e.g. shorter reaction time) for incongruent trials following an already incongruent one. The role of higher-order cortices in such between-trial adjustments is well documented, however, a specific involvement of the primary motor cortex (M1) has seldom been questioned. Here we studied changes in corticospinal excitability associated with the conflict-adaptation process. For this, we used single-pulse transcranial-magnetic stimulation (TMS) applied between two consecutive trials in an interference flanker task, while measuring motor-evoked potentials (MEPs) after agonistic and antagonistic voluntary movements. In agonist movement, MEP amplitude was modulated by recent movement history with an increase favoring movement repetition, but no significant change in MEP size was observed whether a previous trial was incongruent or congruent. Critically, for an antagonist movement, the relative size of MEPs following incongruent trials correlated positively with the strength of behavioral adaptation measured as the degree of RT shortening across subjects. This post-conflict increase in corticospinal excitability related to antagonist muscle recruitment could compensate for a potential deleterious bias due to recent movement history that favors the last executed action. Namely, it prepares the motor system to rapidly adapt to a changing and unpredictable context by equalizing the preparation for all possible motor responses.

## Introduction

**Figure 1:**
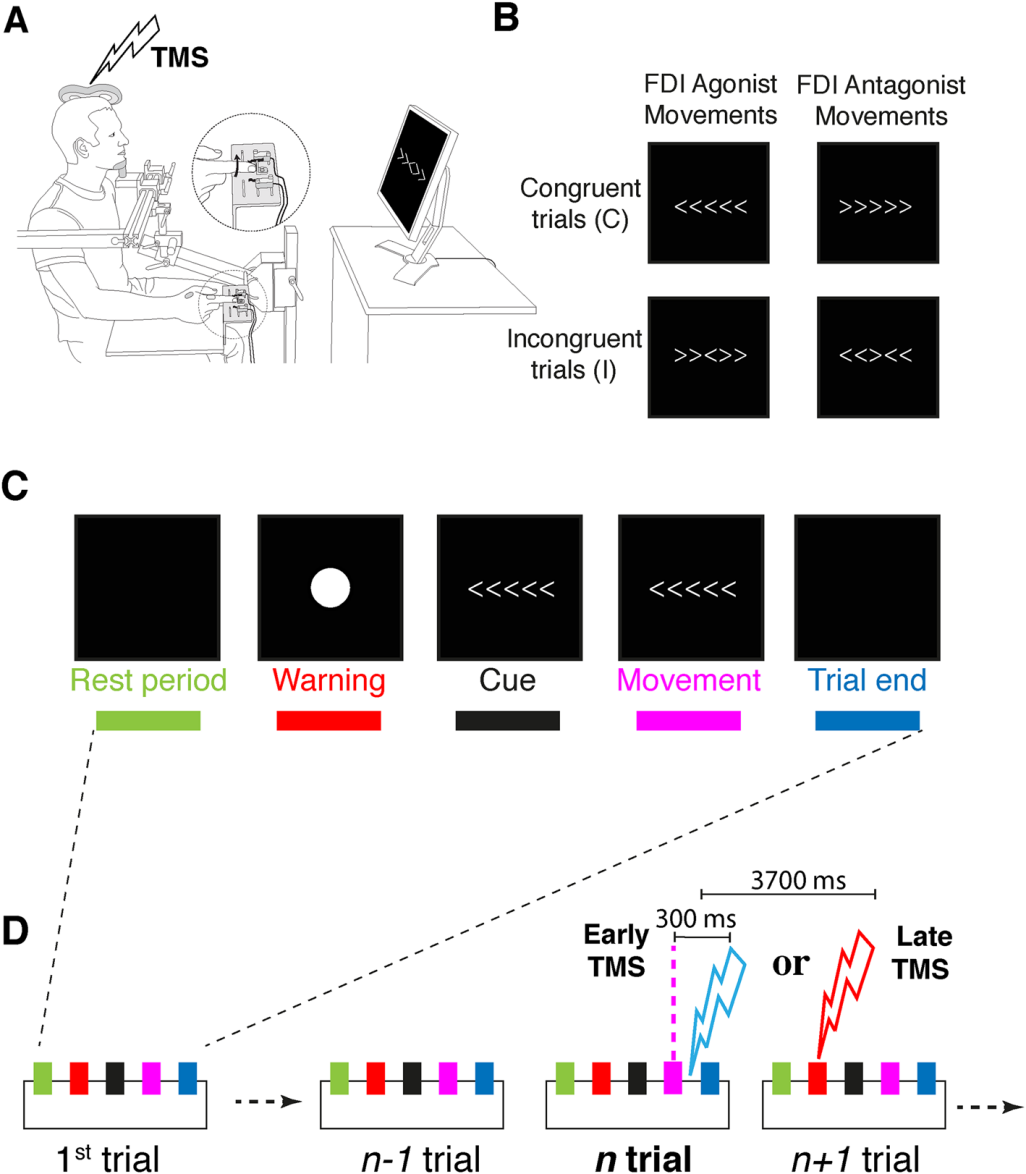
Experimental design. (**A**) Subjects responded to the task by either flexing or extending the index finger of their right hand (inset) in response to the appearance of a visual stimulus. Single-pulse transcranial magnetic stimulation (TMS) was applied over the left primary motor cortex, and EMG activity was recorded from the 1st dorsal interosseus muscle (FDI) of the right hand. (**B**) Experimental conditions of the flanker task involving the 4 possible stimuli. (**C**) Each trial began with a 1000 ms rest period, followed by a 1000 ms warning stimulus (white circle on black screen, 5 cm diameter) displayed at the center of the screen. A 500–800 ms delay then preceded presentation of the cue arrows to which the subject had 2000 ms to respond. The cue arrows were displayed until movement onset and each trial ended approximately 1600 ms after movement completion. (**D**) During the task, single-pulse TMS was applied relative to the movement in the current (*n*) trial at 2 different latencies (early, at 300 ms post-movement and late, at 3700 ms post-movement). Note that, effectively, the late TMS for the current trial fell at the beginning of following (*n* + 1) trial. TMS pulses of the two latencies were never applied within the same trial or between two consecutive trials in order to avoid a potential cumulative effect of stimulation.

Even when we try to control our environment, unpredictable events or sudden changes are impossible to avoid and action decisions must consequently be adjusted in order to reach our initial goal. This adaptive capability is a central feature of voluntary (or goal-directed) behavior and is thought to rely on the cognitive or executive control function process^1^. This process necessarily engages two complementary aspects: (1) evaluation in order to detect the occurrence of a ‘change’ in the environment, and (2) the subsequent implementation of behavioral adaptation mechanisms^1^. One of the most studied models of behavioral adaptation that supports the existence of a cognitive control of action, along with post-error slowing^2^, is so-called post-conflict behavioral adjustment. This can be observed during the performance of an interference-task in which congruent (C trial) and incongruent (I trial) stimuli (Fig. 1A and Table 1) are successively presented to subjects instructed to elicit an immediate motor response. The latter is characterized by a shortening in reaction time (RT) to an incongruent stimulus when another incongruent situation was just experienced (iI trials), in comparison with an incongruent stimulus following a congruent one (cI trials)^3^.

**Table 1:** The 16 different stimulus transitions employed in the flanker task.

Stimulus		Trial condition		Repetition (+) vs change (−) Movement *(trial* n)	FDI role *(trial* n)	Figure 4 panel correspondance
Trial *n−1*	Trial *n*	*n−1*	*n*			
<<<<<	<<<<<	c	C	+	Ago	A
<<<<<	>> <>>	c	I	+	Ago	
>> <>>	<<<<<	i	C	+	Ago	
>> <>>	>> <>>	i	I	+	Ago	
>>>>>	<<<<<	c	C	−	Ago	B
>>>>>	>> <>>	c	I	−	Ago	
<<><<	<<<<<	i	C	−	Ago	
<<><<	>> <>>	i	I	−	Ago	
>>>>>	>>>>>	c	C	+	Antago	C
>>>>>	<<><<	c	I	+	Antago	
<<><<	>>>>>	i	C	+	Antago	
<<><<	<<><<	i	I	+	Antago	
<<<<<	>>>>>	c	C	−	Antago	D
<<<<<	<<><<	c	I	−	Antago	
>> <>>	>>>>>	i	C	−	Antago	
>> <>>	<<><<	i	I	−	Antago	

Letters at left refer to the congruent or incongruent condition of the previous (n−1; c or i) and the current (n; C or I) trial. Repetition or change in movement direction in transitions between trial pairs are indicated by (+) and (−), respectively. Also indicated is the agonist (Ago) or antagonist (Antago) role of the FDI muscle in the current (n) trial. The column at right indicates the trials used to calculate the CSE indexes presented in Fig. 4, with letters A,B,C,D corresponding to each figure panel.

**Figure 2:**
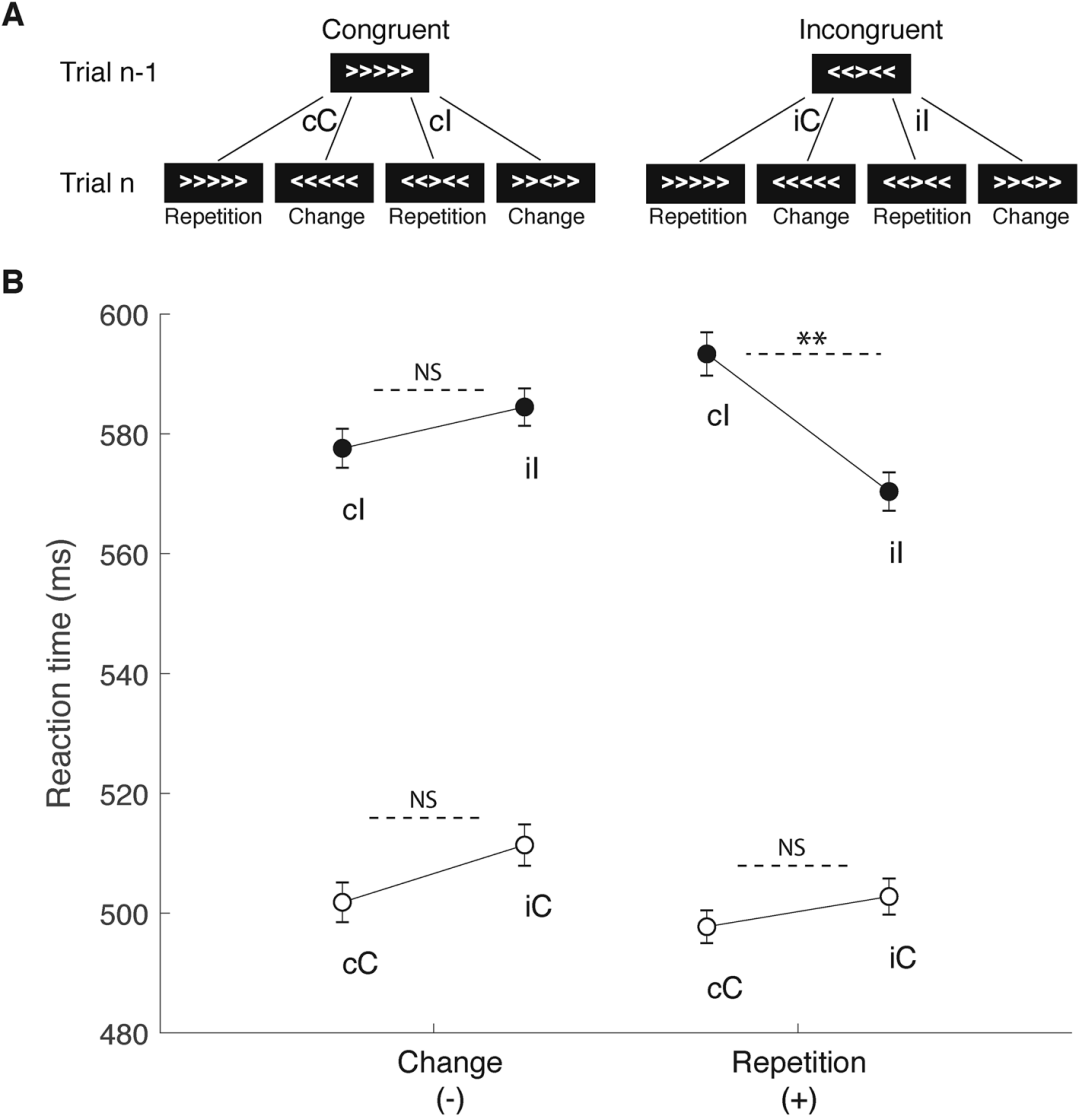
(**A**) examples of trial-to-trial transition in the flanker task. (**B**) Behavioral measurements for all (flexion or extension) movements. Values are mean (± SE) reaction times (RTs) during trial *n* as a function of congruency of the stimulus in trial *n* (C or I) and trial *n−1* (c or i). The data are grouped according a change (−) or repetition (+) of stimulus direction between trial pairs. A significant conflict-adaptation effect (iI < cI) was found only in the case of stimulus repetition but not stimulus change. **p < 0.01 (see also Table 1).

However, it has also been shown that repetitions of the same movement/action (Fig. 2A and Table 1) can produce trial-to-trial adaptations that could conceal a use-dependent effect in post-conflict behavioral adjustment^4^. This repetition effect has long been considered a potential caveat in adaptation studies and although generating vigorous debate^5,6^, it has never been considered alone to be a potential limitation to the adaptive process. Indeed, the well-known bias resulting from the repetition of similar voluntary movements could be potentially deleterious in an uncertainty context where the next action to select is not fully predictable^7,8^. Hence, such a bias in action selection that helps to improve performance in a repetitive task or context could affect the efficacy (speed or even accuracy) in choosing a different option. In this respect, several studies have pointed out the role played by the primary motor cortex in such a use-dependent effect^7,9,10^, as well as a preeminent role of the anterior midcingulate cortex (aMCC) and prefrontal cortices^11^ in the enhancement of attentional resources that ensure a better processing of the relevant feature of the task at hand^12^. Consequently, a type of hierarchical organization has been described in which the top-down control is implemented in part via the anterior midcingulate cortex (aMCC) and prefrontal cortices^11^, whereas the primary motor cortex (M1) is responsible for the final motor selection. In this context, it is noteworthy that the brain regions mentioned above are anatomically related since elements of the aMCC project directly to the M1^13,14^. However, previous reports proposed that the M1 is more than the simple relay for motor execution^15–17^. From this perspective, M1 is considered not only the last cortical relay before reaching the spinal motor neurons but also an integrative area within which cognitive information can still influence decision making^18^. However, there is no direct evidence that M1 is also involved in the trial-to-trial adaptive process that accounts for the post-conflict behavioral adjustment.

If the M1 is involved in such short-term adaptive processes, then it should integrate cognitive information that influences not only a current action but also an upcoming one. Consequently, its activity should be different with respect to, as well as being shaped by, the previous trial condition. Based on the competition between potential actions that is known to occur within the motor system^19,20^, we hypothesized that this modulation of M1 activity should affect neurons tuned to the actual selection of actions. Practically, this implies that this modulation is directly related to the cognitive condition of the previous trial, whatever the movement history.

Here, we used a modified version of the Eriksen flanker task for studying response conflict since this task has been well characterized (Fig. 1A–C; “Methods”)^4,17,21,22^ and thus allows data comparisons to be made with previous studies. Practically, we stimulated the M1 (either early or late) after the subject had completed a movement response in a given trial and before the selection of another response for a subsequent trial (Fig. 1D). Therefore, we postulated that if neural activity in M1 reflects the adaptive post-conflict process, changes in cortico-spinal excitability (CSE) should occur after incongruent vs congruent trials.

## Methods

### Participants

Fourteen subjects (7 females), mean age 23.5 (± 3.2) participated in the experiment. All participants had normal or corrected-to normal visual acuity, were right-handed according to the Edinburgh Handedness Inventory^23^ and were free from any contraindication to transcranial-magnetic stimulation (TMS)^24^. The experimental procedure was approved by the ethics committee (“Comité de protection des personnes sud-ouest et outre mer III”; approval number No 2013A01444936), and was carried out in accordance with the principles of the revised Helsinki Declaration^25^. All subjects gave written informed consent prior to the experiment.

### Task design

The subjects performed the Eriksen flanker task^17,21^, responding by either flexing or extending the index finger of their right hand to the appearance of a visual stimulus. Participants were seated in a comfortable chair, their eyes at a distance of 75 cm from a computer monitor. Their wrist was constrained during the experiment, and subjects were specifically instructed to perform the task only with their right index finger without moving any other joints. A custom-built apparatus supported the right forearm and hand. It was equipped with infrared position sensors at the central (rest) position of the index finger as well as at the left and right peripheral position (to monitor respectively left and right movements in response to the target). This allowed detection of both the finger release from the rest position and the actual choice (button pressed) made by the subject. The task was an arrow version of the classical Eriksen flanker task (Fig. 1B). Two different conditions (Fig. 1B and Table 1) were used in the task: in the *congruent* condition, four flanker arrows appeared beside the central arrow, pointing in the same direction (<<<<< or >>>>>). In the *incongruent* condition, the flanker arrows pointed in the opposite direction (flexion for >> <>> and extension for <<><<). In both congruent and incongruent conditions, subjects were instructed to respond by flexing or extending their index according to the central arrow direction. All stimuli were presented in white on a black background. Task stimuli were manipulated and presented in a pseudo-random order in order to satisfy the following parameters: (1) congruent (C) and incongruent (I) trials were presented in equivalent proportions; (2) similar number of trials for movement direction demanded for responding to the central arrow (left pointing arrow = first dorsal interosseous (FDI) muscle contraction (FDI agonist) or right pointing arrow = extensor indicis (EI) muscle contraction (FDI antagonist movement)); (3) similar number of trials regarding the movement history (repetition, i.e. two successive movements in the same or changed direction); (4) similar number of trials regarding the cognitive condition sequence effect (congruent followed by congruent: cC; incongruent followed by congruent: iC; congruent followed by incongruent: cI; incongruent followed by incongruent: iI). Each trial (Fig. 1C) began with a 1000 ms rest period, followed by a 1000 ms warning stimulus (white circle on black screen, 5 cm diameter) displayed at the center of the screen. Then a 500–800 ms delay preceded the presentation of the cue arrows. The subject had 2000 ms to respond. Cue arrows were displayed until the movement onset and each trial ended approximately 1600 ms after movement completion.

### EMG and MEP recording

**Figure 3:**
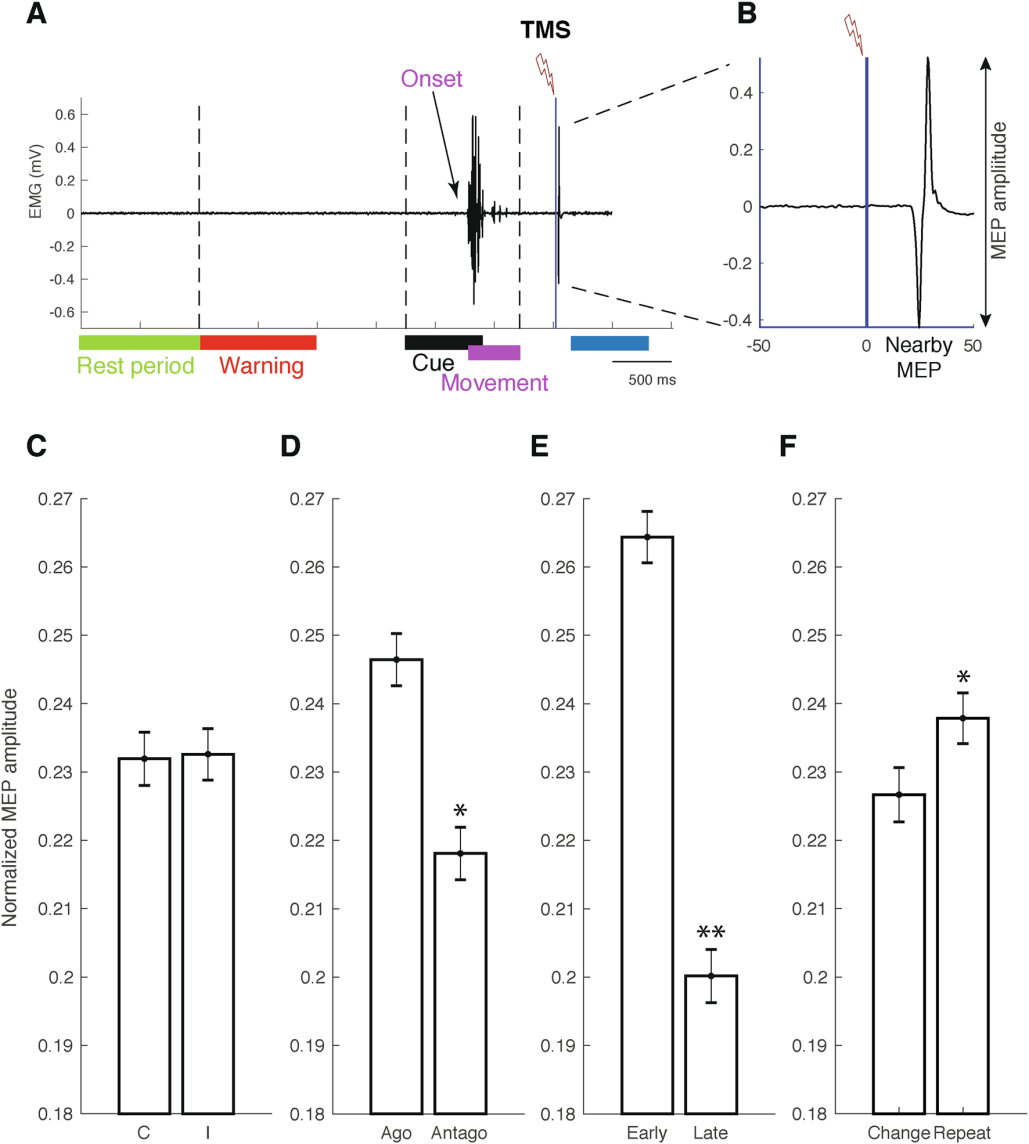
(**A**) Example of EMG activity recorded in a trial with agonist FDI movement and the MEP evoked by an early TMS after the voluntary muscle contraction. (**B**) Zoom of the MEP whose amplitude was used as a measure of corticospinal excitability. (**C**–**F**) Normalized MEP amplitudes as a function of the congruent or incongruent cognitive condition (**C**), agonist or antagonist movement direction (**D**), early or late TMS timing (**E**) and movement history (change or repetition) (**F**). Note the lack of altered post-movement CSE modulation as a function of cognitive condition in trial n (**C**), but an increase in CSE when an agonist movement had just been executed (**D**). This effect persisted after movement execution, (**E**, left), but had declined significantly by the time of the late TMS (**E**, right). CSE also increased after a succession of identical target/response associations (**F**), indicating that despite a diminishing CSE over time, a medium-term influence of movement history remained. **p < 0.01; *p < 0.05.

Surface electromyographic (EMG) recordings were made from the FDI (a finger flexor) and the EI of the right hand. EMG activity was acquired by a Trigno Wireless EMG Systems amplifier (DELSYS Inc., Boston, MA, USA), amplified by a factor of 909, band-pass filtered (Bandwidth 20 ± 5 Hz, > 40 dB/dec), digitized on line (rate 2 kHz), and later rectified and filtered in order to obtain the linear envelope of the signal. The movement onset for index flexion (leftward movement) and extension (rightward movement) was detected by the voluntary contraction onset of FDI and EI muscles, respectively, using MATLAB programming and verified visually for each trial (Fig. 3A): This onset detection algorithm is based on the main threshold selection strategy. The threshold value corresponds to the mean value of the EMG signal ± 2 SD (in mV) recorded during the rest period. The time point after the visual stimulus presentation where the EMG signal first exceeded this threshold level for at least 20 sampling points (10 ms at 2 kHz) indicated EMG activity onset time. A systematic comparison with onset detection values provided by an infrared position sensor at the central (rest) position of the index finger was also performed. Because the EI is a deep lying muscle, electrodes over this muscle recorded both extensor and flexor activity. Nevertheless, it allowed us to precisely detect extension onset, as verified by systematic comparison with the position sensor displacement onset. Indeed, while flexion was recorded by both the FDI and EI electrodes, extension involved only EI contraction. Our analyses of motor evoked potentials (MEPs) focused on the FDI muscle, and flexion and extension are hereafter referred to as agonist and antagonist movements, respectively. In order to avoid any significant modification of MEP amplitudes due to background noise in the EMG recordings, trials in which muscular pre-activation was greater than 100 μV within the 500 ms window immediately preceding the TMS pulse were discarded.

### TMS testing

A figure-of-eight coil (Double 70 mm Coil, Magstim Company Ltd, Whitland, Dyfed, UK) was used to stimulate the M1 over the left hemisphere. The coil was connected to a Magstim stimulator (Magstim Super Rapid^2^, maximum output 1.2 T at the surface of the coil, Magstim Co. Ltd. Whitland, Dyfed, UK) and was held tangentially on the left hemiscalp with its handle pointing backwards at an angle of about 45° from the midsagittal axis. The motor hotspot was defined as the optimal (minimum TMS intensity) scalp position (OSP) for the induction of MEPs of approximately 1 mV peak-to-peak amplitude at rest (in at least 8 of 10 consecutive trials). The OSP was obtained by moving the coil in approximately 0.5 cm steps around the subject’s left M1. Stimulation intensity during the recording session was 100% of the resting motor threshold. The mean intensity of a single TMS pulse needed to evoke a MEP of ~ 1 mV at rest was 58.2% ± 10.6% of stimulator output. Figure 3A illustrates an example of typical EMG activity recorded from the FDI before, during and after the late TMS pulse (Fig. 3B for a zoom on a typical single MEP).

Throughout the experiment, the coil was manually maintained over the hotspot using the Brainsight frameless stereotactic system (Rogue Research Inc, Montreal, Quebec, Canada) to continuously monitor coil placement, with coordinates of each stimulation relative to the hotspot being recorded for post hoc verification. The MEP produced by each stimulation was measured from the FDI muscle of the right hand, and the size of this MEP (peak-to-peak amplitude) was used as a measure of the CSE at the time of the TMS pulse.

The experimental design was divided into four blocks of 160 trials beginning after verbal explanation of the task and the completion of 20 preliminary trials. During each block, the TMS was applied in a pseudo-random order to have an equivalent number of simulated trials across the cognitive condition sequences (cC, iC, cI, iI), movement direction (agonist or antagonist), movement history (change or repetition) and TMS pulse latency (early or late). The TMS pulse was applied over the left M1 at early or late latencies (respectively 300 ms and 3700 ms after movement completion). The early TMS time corresponds to the time taken for the subject to return to resting position, with her/his hand on the position sensor with no residual EMG activity in recorded muscles. The late TMS is more arbitrary since it was aimed at testing the CSE at a distance from the early TMS time. We chose to stimulate during the warning stimulus presentation of the next trial because the subject is in a stable attentional state, without expressing any movement preparation related neuronal activity since information regarding the upcoming action is not yet available.

The mean number of MEPs computed for each correct trial in each of the 32 different experimental conditions (pre-condition (n − 1 trial = c or i) × condition (trial n = C or I) × movement direction × movement history × TMS time) was 11.9 per subject. To check for any effect on CSE by the TMS stimulation per se, we performed 10 TMS stimulations outside the context of the task, at the very beginning and at the very end of each experiment, to obtain the MEP baseline. Importantly, early and late TMS pulses were never applied within the same trial and the mean latency between pulses was 9.3 ± 0.07 s.

### Behavioral measurements

The reaction time (RT) was measured between the cue appearance and the beginning of the voluntary change in EMG activity (Fig. 3A). RTs greater than 1000 ms were considered to be unattended responses and thus were removed from subsequent analysis. For the statistical analysis of RTs, three factors were considered: one corresponding to the cognitive condition according to a given trial’s position (n − 1 and n), and a second factor corresponding to the movement history (change or repetition). Three-way ANOVAs (with the addition of subject number as a third random factor) and *t*-tests with Bonferroni correction for post hoc analysis were used. This analysis was performed in MATLAB (anovan function). We set the significance level for the ANOVAs to *p* < 0.0001 to correct for multiple comparisons, and for the post hoc* t-*tests, to *p* < 0.01. All data are given as means ± standard error (SE). Error rate was defined as the number of error trials divided by the total number of overt responses.

### MEP measurements

Because of the very small number of error trials (0.8 % and 0.09% for the incongruent and congruent trials, respectively), analyses were performed only for correct responses. To minimize the impact of inter-subject variability in MEP amplitudes, we performed a normalization of the raw MEP amplitude values for each subject, which allowed pooling the MEPs from all conditions. Raw MEP sizes were normalized according to the formula:

1 $$\text{Normalized} \; \text{MEP }=\frac{MEPn-MEP{min}}{MEP{max}-MEP{min}}$$

where *MEPn* is the MEP measured on trial *n*, and *MEPmin* and *MEPmax* are respectively the minimum and maximum MEPs measured for that subject.

For the relative MEP amplitude analysis, four factors were again considered: cognitive condition (congruent or incongruent), TMS time (early or late), movement direction (agonist or antagonist) and movement history (change or repetition). Repeated-measure ANOVAs and *t-* test with Bonferroni-Dunn correction for post hoc analysis were used. The significance level for the ANOVAs was to *p* < 0.01 to correct for multiple comparisons and for the post hoc* t*-tests to *p* < 0.05. All data are given as means ± SE and were examined for normality and homogeneity of variance.

## Results

### Behavioral data

A comparison of RTs in the different trial conditions showed the previously observed impact of cognitive condition sequence on the conflict adaptation effect (i.e., longer RTs during cI compared to iI trials Fig. 2; see^4^ : A three-way ANOVA (condition × sequence × subjects) of the RTs under the four cognitive conditions (sequences cC, iC, cI, iI) and the two sequence types (change or repetition)—i.e., the movement history—showed main effect of cognitive condition on the RT (*F*(3,39) = 168.9; *p* < 0.01). Critically, moreover, a significant interaction effect of cognitive condition × sequence (*F*(3, 39) = 10.8; *p* < 0.01) was observed. This was confirmed by post hoc comparison, which revealed that the conflict adaptation effect (cI > iI) was present only for repetition trial RTs (593.3 ± 3.5 vs 570.3 ± 3.1; *p* < 0.0001) and not for the RTs of change trials (577.6 ± 3 vs 584.5 ± 3.1; p > 0.05; Fig. 2). Additionally, the difference between iI trial RTs for repetition and change was statistically significant (*p* < 0.0001). Interestingly, when considered individually, a clear inter-individual difference in conflict adaptation on RT was observed. For the change sequences, although the overall population effect was not statistically significant, 5/14 subjects expressed a clear conflict adaptation in their RTs for cI > iI. For the repetition sequences, 13/14 subjects presented a positive adaptation effect.

### MEP data

The clear difference in RTs between repetition and change trials confirmed that this behavioral protocol was suitable for studying the neuronal basis of the impact of repetition and cognitive condition on conflict adaptation. To this end, we assessed changes in CSE associated with conflict adaptation by measuring the amplitude of MEPs under the different conditions (congruent vs incongruent) and with both types of movement history (repetition or change).

In a next step, the relative MEP amplitude as a dependent variable (see “Methods”, MEP measurement description) was analyzed using a four-way repeated-measure ANOVA (cognitive condition × TMS time × movement direction x movement history) (Fig. 3C–F). There was no main effect of condition for congruent and incongruent trials, since cortical excitability (relative MEP amplitude ± SE) was virtually identical in both conditions (0.23 ± 0.003; *F*(1,4298) = 0.02; *p* = 0.88) (Fig. 3C). In contrast, there was a significant decrease in CSE between early and late TMS times (respectively 0.26 ± 0.003 and 0.20 ± 0.003; *F*(1,4298) = 11.95; p < 0.01) (Fig. 3E) and a significant increase in CSE after agonist movements (0.25 ± 0.003) compared to antagonist movements (0.21 ± 0.003; *F*(1,4298) = 7.34; *p* = 0.01) (Fig. 3D). Finally, concerning the movement history factor for exact target/response repetitions, a TMS applied after a succession of two movements in the same direction (repetition) in response to the same target evoked a significant increase in MEP amplitude (0.24 ± 0.003) in comparison with sequential movements performed during target/response changes (0.22 ± 0.003; *F*(1,4298) = 6.52; *p* = 0.02) (Fig. 3F).

Finally, the inter-individual differences in the behavioral adaptation effect observed across our population of 14 subjects allowed us to examine whether the ‘strength’ (measured by the degree of RT shortening, see below) across subjects of this effect and CSE change were correlated. For this, a CSE index was calculated from relative CSE measurements in incongruent and congruent trials for individual subjects as follows:

2 $$\text{CSE} \; \text{index }=\frac{\text{MEPincongruent }-\text{MEPcongruent }}{\text{MEPincongruent}+\text{MEPcongruent}}$$

**Figure 4:**
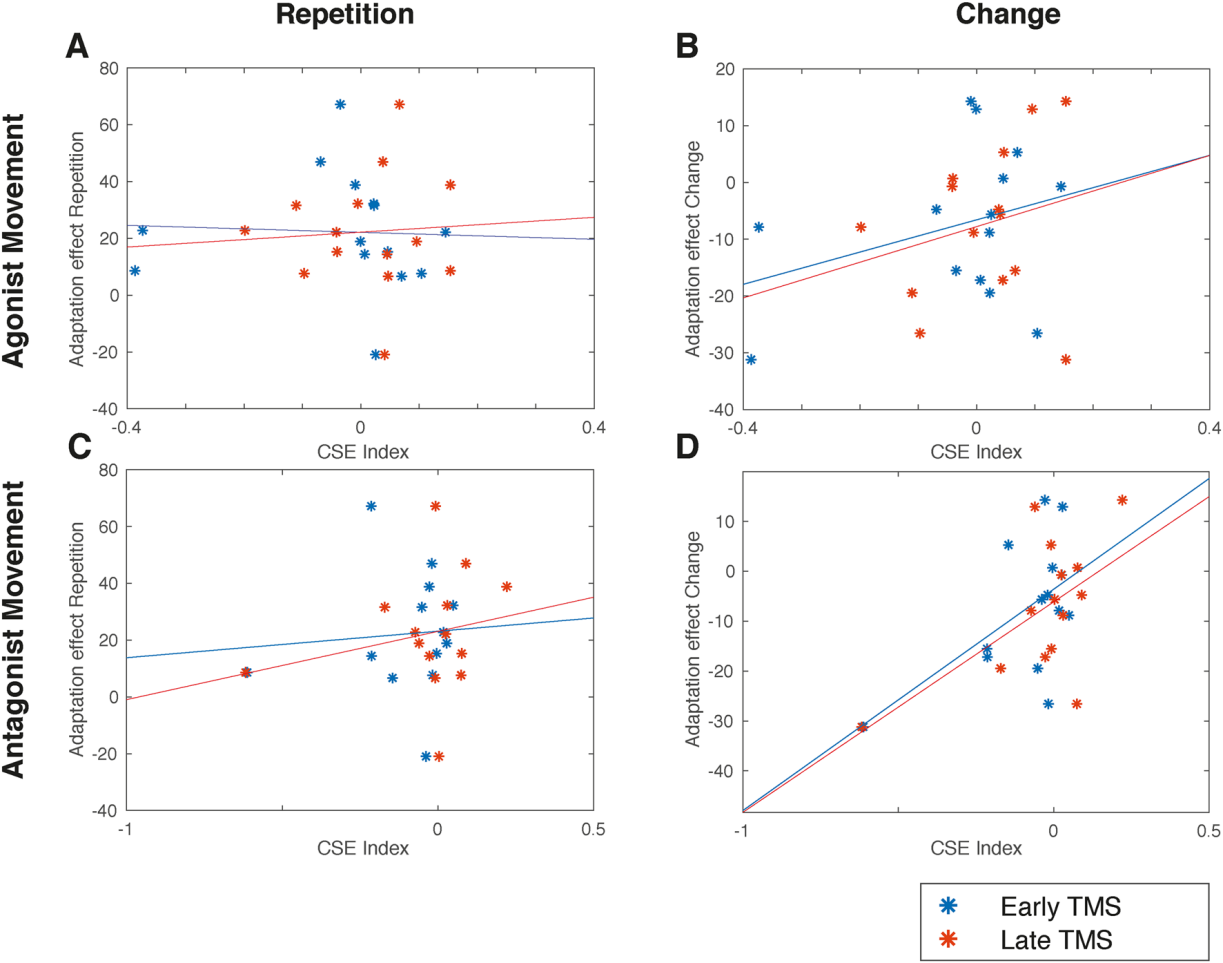
Conflict-adaptation effect (RT for cI – RT for iI trials) as a function of the CSE index (MEPincongruent − MEPcongruent)/(MEPincongruent + MEPcongruent) in each of the 14 subjects. (**A**–**C**) No correlation is evident between agonist or antagonist movement and CSE index in repetition trials (**A**,**C**), nor with agonist movement in change trials (**B**). (**D**) However, the relative strength of the CSE index for the antagonist muscle correlated significantly with the ‘pure’ behavioral adaptation effect (i.e., without repetition) across the subjects. Thus the antagonist CSE index is a reliable predictor of the conflict-adaptation effect for both early and late TMS times (R2 = 0.3 and R2 = 0.36; p < 0.05). See Table 1 for the trial characteristics used to calculate the CSE indexes in (**A**–**D**).

For each subject, we computed eight correlation values between the adaptation effect (RT for cI – RT for iI trials) and the CSE index as a function of repetition, TMS timing and movement direction (Fig. 4 and Table 1). Critically, a significant relationship was found only when the FDI muscle acted as an antagonist (i.e., when the movement direction indicated by the central arrow was towards the right) and only in change trials (i.e., without repetition). In this situation a significant positive correlation between adaptation effect was found for both early (*r* = 0.3, n = 14; *p* < 0.05) and late (*r* = 0.36, n = 14; *p* < 0.05) TMS pulse latencies (Fig. 4D). Thus, subjects exhibiting a larger behavioral adaptation effect also showed an increased primary motor cortex excitability for the antagonist movement direction after an incongruent trial. This in turn suggests that the muscle involved in the non-executed (antagonist) action is primed for a future action when an incongruent condition has just been encountered.

## Discussion

The role of the M1 for movement execution is one of the earliest and more robust findings in neurophysiology^26^. A growing body of evidence points to this motor area also being involved in cognitive aspects of motor function, such as the integration of decision-related information^17,18,27,28^ as well as learning, mental imagery or even error observation^16,29–31^. The present study aimed at exploring M1 changes in excitability or plasticity during the so-called conflict-adaptation effect, characterized by a reduction in error rate and a shortening of reaction time in response to a conflict situation when another conflict situation had just been experienced^3^.

Interestingly, our first analysis including all correctly executed trials failed to reveal a conflict-adaptation effect. Only 5 out of 14 subjects exhibited a shortening of RTs for iI trials compared to cI trials. However, when we took into account movement history in analyzing separately the repetition trials (successive trials with identical stimulus-response associations) we found a significant conflict-adaptation effect for 13 from 14 subjects. This result thus confirms a previous report that stimulus-specific priming can alone account for the adaptation effect, which could be linked to a ‘memory-based’ priming^4^.

Although evaluating the current controversy concerning the conflict adaptation effect is beyond the scope of this paper, it is interesting to note that in studies where the repetition priming effect was observed, the presentation of visual stimuli lasted until a decision was made and subjects were not under any time constraint to respond^4^. Conversely, studies that used either a shorter exposure to visual stimuli or a time pressure for the decision process failed to observe a priming effect following repetition^3,22,32^. In our study, stimuli were displayed until the initiation of action and we did not impose any time constraint on our subjects, which raises the possibility that a speed-accuracy tradeoff dictated by the experimental design has an impact on the effect of repetition. Additional studies are therefore needed to determine the consequences of the speed-accuracy tradeoff for the priming effect of repetition on conflict adaptation.

Among the several factors that influence voluntary movements, sequentiality is often used in the laboratory to study training-related improvement of performance, such as a shortening of RTs and a reduction of error rates^3^. In this context, applying TMS after the execution of movement allowed us to probe CSE between two successive decisions, when information regarding the next action (cognitive condition and movement direction) were not yet available. In this case, MEP modulation could be interpreted as immediate changes of CSE attributable to past experience. Furthermore, the inter-individual variability in performance gave us an opportunity to analyze a wide range of values of the adaptation effect, a process that is neither stereotyped, nor fixed or absolute. The CSE related to agonist muscle contraction after both incongruent or congruent trials was not modulated either early or later after the movement execution. However, the behavioral adjustment effect (namely the Gratton effect) considered on an individual subject basis was correlated with the CSE index of the antagonist muscle, indicating that a larger conflict-related change in CSE would result in a higher probability of behavioral adaptation during the subsequent trial (trial n). Accordingly, we found that in a two choice interference forced task, the speeding up of RTs in successive incongruent trials depended on a flattening (i.e. a reduced activity differential) of excitability between the two movement directions, caused by an increased excitability of the M1 region controlling muscles involved in the unselected movement option. As a consequence, the movement-history bias for the upcoming action would be correspondingly reduced.

Several studies have emphasized the contribution of movement-history bias, characterized by an increased probability to select the same movement as the previous one and sustained by a change in synaptic efficacy^7,33,34^. In our experiment, this conclusion is supported by our finding that the MEP from the muscle just activated has a greater amplitude than that of the antagonist muscle, whatever the cognitive condition, thereby favoring repetition of the same movement. This result is also in accordance with the behavioral benefits of practice on the learning process^7,9^. Interestingly, even though movement history bias is considered to be important for skill acquisition^7^, it could also underlie a potential limitation to an adaptive process since it favors action repetition even in situations that are not appropriate. Consequently, part of the executive process could be slowed-down when an alternative action is required. In contrast, equalizing the probability of potential responses could be an adaptive solution to preventing unduly prioritizing one action over another. In an experimental context of uncertainty, where all options are equiprobable, also supporting an alternative response has a clear behavioral advantage. The nature of the Flanker task itself is particularly well adapted to favoring this type of response since, in the conflict condition, the relevant stimulus is flanked by distractors that encode the potential alternative response. In any case, this process completes the range of adaptive mechanisms, some of which involve a top-down attentional process like that leading to the suppression of inappropriate motor representations when the conflict context is predictable^35^. Overall, our results are in accordance with a previous study showing that the post-error slowing, another well-known adaptative behavioral effect that occurs after a subject has just committed an error, is in part prevented by a subsequent (450 ms post- action) decrease in motor cortex excitability involved in the erroneous response^36^. In this context, nevertheless, it is noteworthy that the aMCC, which projects directly to the M1, is considered to be the main cortical locus for conflict monitoring^37^ and is also involved in the choice of an alternative action when the expected outcome of a former action is not obtained^38,39^. Thus, it appears from previous and our present work that both top-down (attentional) and bottom-up (action) parameters participate in the decision process depending on a preceding action, and that the aMCC and M1 could play a complementary role in this process. A likely advantage of the ‘automatic’ modulation of antagonist CSE is that it would be less sensitive to external disturbance. Further studies are necessary to confirm this hypothesis, as well as the dependency on higher order motor cortices like the aMCC.

As a whole, different types of control processes are involved synergistically in efficiently adapting motor actions to a complex and changing environment^40^. It is therefore likely that a continuum exists between the relatively simple mechanism described in this study and more complex cognitive functions like conflict monitoring^37^; whose predominance could effectively conceal the former’s contribution to ensuring adaptive behavior.
